# Culturally and structurally competent approaches to health research with Black communities in Atlantic Canada: a rapid review

**DOI:** 10.24095/hpcdp.45.4.04

**Published:** 2025-04

**Authors:** Joshua Yusuf, Emma Stirling-Cameron, Keisha Jefferies, Bamidele Bello, Chelsa States, Barbara-Ann Hamilton-Hinch

**Affiliations:** 1 School of Health and Human Performance, Dalhousie University, Halifax, Nova Scotia, Canada; 2 Healthy Populations Institute, Dalhousie University, Halifax, Nova Scotia, Canada; 3 School of Population and Public Health, University of British Columbia, Vancouver, British Columbia, Canada; 4 School of Nursing, Dalhousie University, Halifax, Nova Scotia, Canada

**Keywords:** Black Canadians, racism, health inequity, cultural competency, Atlantic Canada

## Abstract

**Introduction:**

Anti-Black racism is deeply entrenched in Canadian institutions and has deleterious impacts on Black populations. Black populations have resided in the Atlantic region since the late 17th century. Despite longstanding histories, Atlantic Black populations face significant inequities, including the highest rates of child poverty among Black children across Canada. Community consultations in Atlantic Canada have highlighted a desire to bring attention to these health inequities. The purpose of this review was to identify existing literature pertaining to Black health research in Atlantic Canada and highlight culturally appropriate practices.

**Methods:**

The search strategy was developed with a librarian and focussed on health research pertaining to Black populations in the Atlantic provinces of Canada, covering eight databases. All articles were imported into Covidence for screening, with independent reviewers assessing titles, abstracts and full texts.

**Results:**

Forty-seven studies met the inclusion criteria. Findings demonstrated the pervasiveness and impact of racism, the importance of community engagement as a key cultural consideration and the adoption of participatory action research frameworks as culturally appropriate.

**Conclusion:**

This review revealed opportunities for improving Black health research in Canada’s Atlantic provinces. Future research warrants attention to this region and the use of culturally and structurally appropriate research approaches and methods. Recommendations include improved education on Black history and a training module within existing ethical guidelines for culturally and structurally competent research with Black communities.

**Highlights**

- Atlantic Black populations face significant health inequities, and there is a need to identify and to develop culturally appropriate practices to help address these inequities.
- The rapid review identified 47 studies on Black health in Atlantic Canada.
- The findings underscore a significant gap in culturally and structurally competent Black health research in Atlantic Canada.
- A culturally and structurally competent research base begins with all researchers completing at least one module on ethical research with Black populations.

## Introduction

The systematic inequities facing Black people have persisted since Canada’s colonization, but police brutality, global anti-racism protests and a pandemic that disproportionately affected Black communities have brought anti-Black racism into prominence.^1^-^4^ Anti-Black racism in Canada is defined as “policies and practices rooted in Canadian institutions, such as education, health care and justice, that mirror and reinforce beliefs, attitudes, prejudice, stereotyping and/or discrimination towards people of Black-African descent.”^5^ The legacy of colonialism, the Trans-Atlantic slave and historic segregation have created a social ecosystem that disproportionately disadvantages Black Canadians. This has manifested in disproportionately high rates of poverty in Black families,^6^ limited access to education^7^,^8^ and inequitable access to healthcare for Black Canadians, resulting in significant health and social disparities.^9^,^10^ Despite growing recognition of the magnitude and pervasive nature of anti-Black racism and the health inequalities it has created, comprehensive, empirical research reporting on the health outcomes of Black Canadians remains limited. 

Eurocentric research institutions have a long and problematic history of perpetrating harm and excluding racialized populations from research, policy and practice. The legacy of misappropriated findings, experimentation and the morbid maltreatment of Black bodies and communities (e.g. the Tuskegee Syphilis Study) have had lasting, intergenerational impacts.^11^ Studies have found that people of African descent are more likely than age-, education- and gender-matched White people to believe that research findings will be used to reinforce negative stereotypes^12^ or that the research itself will expose them to unnecessary risks.^13^,^14^ Contemporary research and research methodologies are often exclusionary and continue to include majority White, English-speaking, affluent participants.^13^,^15^ Investigators themselves have been known to assume that people of African descent will not consider participating in research, and do not put in the time or effort to make studies inclusive and safe.^16^,^17^The problematic categorization of participants by race or ethnicity without context has contributed to the perpetuation of negative stereotypes about certain racial groups.^18^ Many standardized measures, interventions and assessments have been designed using majority-White participants and may not be appropriate to use with Black or other racialized people.

Canada has a growing and diverse Black population. As of 2021, over 1.5 million Black people were residing in Canada, comprising 4.3% of the national population.^19^ This number is set to double by 2041, with new immigrants and refugees arriving from Africa and the Caribbean annually.^20^ Despite Canada’s multicultural identity, discrimination against Black people is deeply entrenched and normalized in Canadian institutions, policies and practices, and is often not noticed by non-Black folk.^21^ In fact it has been reported that anti-Black structural racism was present in most of Canada’s core institutions (e.g. health, criminal-legal, education), with ongoing negative impacts causing further marginalization for Black communities.^21^

Stigma and discriminatory maltreatment are experienced by Black Canadians in intersecting ways across individual, institutional and systemic dimensions. This shapes access to social and economic resources that promote health and wellness (e.g. food, housing, education and employment) contributing to chronic stress, with devastating implications for Black people’s physical and mental health. The Public Health Agency of Canada has found significant gaps in the lifespan of Black men and women: White, university-educated men had life expectancies 14.2 years higher than those of Black men without a high school education; and White, university-educated women had life expectancies 10.3 years higher than those of Black women without a high school education.^9^,^22^ Black Canadians were significantly more likely than White Canadians to report diabetes and hypertension.^23^,^24^

Atlantic Canada is a geographic region of Canada that comprises four provinces: New Brunswick (NB), Nova Scotia (NS), Prince Edward Island (PE) and Newfoundland and Labrador (NL). Atlantic Canada is home to a historic Black community known as the African Nova Scotians (ANS) or indigenous African Nova Scotians.^25^ This distinct population has a unique history, with an ancestral lineage dating back to the late 17th century. After that time, thousands of formerly enslaved Black Loyalists arrived in Nova Scotia in 1783, followed by the Jamaican Maroons and other Caribbean immigrants in the 19th and 20th centuries.^26^ In contrast to other regions, these historic Black populations have predominately resided in Atlantic Canada for over four hundred years and have a history and culture specific to the region. This review uses the term “Black” as a collective term for all people of African descent, but recognizes the greater discussion that needs to continue regarding culturally appropriate identifiers.^27^

Though ANS have resisted extensive racial and colonial violence, centuries of maltreatment have contributed to intergenerational trauma, health inequalities and barriers to healthcare access that remain today.^1^,^28^ For example, the Atlantic provinces have reported the highest rates of child poverty among Black children in the country (40% in NS, 41% in NL, 37% in NB and 33% in PE), compared to the non-Black national average of 17%.^29^ Moreover, Black men in Nova Scotia are six times more likely to experience street checks by police than their White counterparts. Additionally, anti-Black racism in the Nova Scotian education system has affected African Nova Scotian children aged as young as 18 months.^7^,^30^,^31^

Community consultations with ANS and other Black communities in Atlantic Canada have highlighted a desire to bring attention to the health inequities facing their communities. Yet, they also report mismanagement of Black health data and the over-researching of their communities. This is consistent with existing evidence documenting how the researchers and health practitioners have caused undue harm to Black communities through insensitive, inappropriate and unethical research.^11^,^14^,^32^ Common examples of repeated and ongoing harm in research include reporting research results in a manner that reinforces negative stereotypes, failing to consult with communities consistently and meaningfully and conducting research that does not align with community needs. While the renewed awareness of anti-Black racism is welcomed, novel research conducted with Black communities must be conducted in a way that promotes their best interests and safety. 

The purpose of this review was to identify existing literature pertaining to culturally and structurally competent Black health research in Atlantic Canada.The objectives of this rapid review were twofold: (1) to identify and document available literature pertaining to Black health in Atlantic Canada; and (2) to describe the research topics, methodologies, methods and reporting techniques employed in studies that examined Black health in Atlantic Canada. The findings of this review will be useful for informing future research with Black communities in and beyond Atlantic Canada. The concept of culturally and structurally competent research was recommended to us by a Black health community organization. The term combines the familiarity of cultural competence (i.e. sensitivity and responsiveness to racial, ethnic, gender-based and sociodemographic differences and preferences) with the less familiar structural competence (i.e. sensitivity and responsiveness to the impact of forces at the societal, policy, socioeconomic and individual levels).^33^ Thus, culturally and structurally competent research is sensitive to both Africentric ways of knowing and the macrosystems that influence Black populations.

## Methods

A rapid review was conducted to develop timely and culturally adept synthesis of the current state of research on Black health in Atlantic Canada. This review was conducted in accordance with guidelines outlined by Tricco et al.^34^ The search strategy was developed in collaboration with a health science librarian at Dalhousie University. The search was conducted in July 2022 and was performed in eight databases: MEDLINE, Embase, CINAHL, Cochrane, PsycInfo, Scopus, Sociological Abstracts and Social Services Abstracts (full search strategy available on request from authors). The criteria for inclusion followed the mnemonic PCC—representing the population, concept and context.

***Eligibility criteria***

**Population**

While ANS constitute a significant portion of Black people in Atlantic Canada, this review included studies with participants of African descent. Search terms included Black, Black-Indigenous, African Nova Scotian, Black Scotian, African descent, Black descent, African. Studies that included multiple populations in Atlantic Canada alongside an analysis of Black populations were included; however, only the sub-analysis of Black Canadians in the Atlantic region was considered.

**Concept**

The concept under study was health as it relates to Black communities in Atlantic Canada. The World Health Organization’s definition of health is “a state of complete physical, mental and social well-being, and not merely the absence of disease or infirmity.”^35^The review includes social determinants of health, referring to the range of personal, social, economic and environmental factors that influence population health, such as socioeconomic status, racism, working conditions, physical environments and access to healthcare.^36^

**Context**

This review considered studies that explored the health of people of African ancestry in the four provinces of Atlantic Canada (New Brunswick, Nova Scotia, Prince Edward Island and Newfoundland and Labrador). The total population of the Atlantic region is 2.41 million.^37^

***Types of sources***

This rapid review considered studies that utilized a variety of research designs, encompassing both qualitative and quantitative data. These included experimental and quasi-experimental study designs, analytical observational studies, descriptive observational study designs, phenomenology, grounded theory, ethnography, and participatory action research. Commentary, opinion and perspective articles were also included in an effort to capture a breadth of research and varying ways of disseminating research. Evidence synthesis articles were not included but reference chaining was used to identify articles for inclusion.

***Screening, study selection and extraction***

All articles were imported into Covidence (Veritas Health Innovation, Melbourne, AU), an online evidence synthesis tool, and were assessed in detail against the inclusion criteria by two independent reviewers. Any disagreements that arose between the reviewers at each stage of the selection process were resolved through discussion or with a third reviewer. The data extraction process was carried out in Covidence. For consensus purposes, two independent researchers extracted the data. The data extraction tool contained fields for purpose and objectives, location of study, population, health dimension, research design, theoretical framework used, data collection and analysis methods, key findings, community engagement techniques, cultural considerations and recommendations. 

## Results

**Figure 1:**
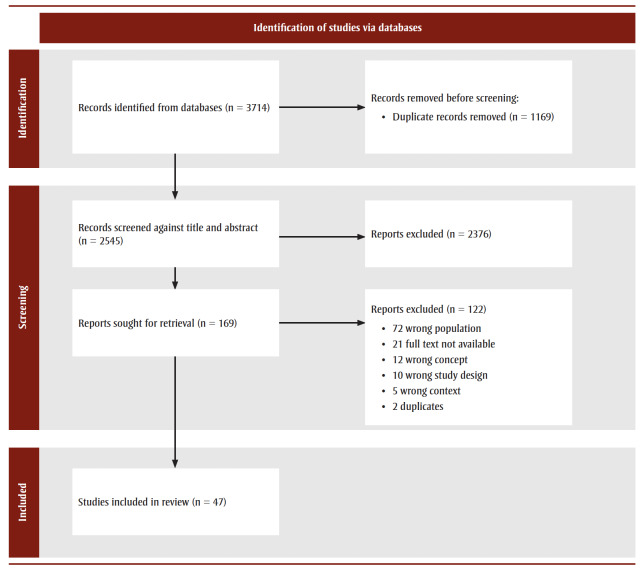
PRISMA chart outlining the stages of the rapid review process and number of sources retrieved and selected for each stage

Three thousand, seven hundred and fourteen (3714) articles were retrieved, and 1169 duplicates removed. Two thousand, five-hundred and forty-five (2545) titles and abstracts were screened by two independent reviewers for assessment against the inclusion criteria. A total of 169 full text articles were reviewed. The results of the search are presented in a Preferred Reporting Items for Systematic Reviews and Meta-analyses Extension for Scoping Reviews (PRISMA-ScR) flow diagram (Figure 1).^34^

This rapid review included a total of 47studies. Two of the studies were published prior to the year 2000, 27 were published between 2000 and 2014 and 18 were published from 2015 onward. Of the studies that met the inclusion criteria, almost all were from Nova Scotia (n=44).^25^,^38^-^80^ Only one study from each of New Brunswick,^81^ Prince Edward Island^82^ and Newfoundland and Labrador^83^ met the inclusion criteria.

Findings are presented in three sections that cluster the stages of the research cycle. The first section, “Designing and implementing research with Black populations,” outlines frequently used approaches and theoretical models, and common features of the target populations, recruitment and data collection and analysis. The second section, “Findings and recommendations of the available research,” describes common findings and recommendations of included studies. Finally, section three, “Considerations outside of Eurocentric approaches to research,” highlights the importance of considering culture and community engagement in research with Black populations. Each section provides a narrative synthesis of extracted data.

***Designing and implementing research with Black populations***

Included studies focussed on varying areas of health and included mental health,^40^,^45^,^49^,^55^,^58^,^78^,^80^,^82^ sexual and reproductive health,^43^,^46^,^54^,^56^,^57^,^59^,^65^,^71^ racism,^39^,^41^,^47^,^52^,^68^,^69^,^71^,^81^ food security^42^,^66^,^67^,^79^ and palliative care.^62^,^75^,^77^ Table 1 provides an overview of research approach; research design; theory, frameworks or models; recruitment and sampling; data collection; and data analysis. Research approaches designated as “other” in Table 1 include articles that do not fall under the traditional qualitative-quantitative dichotomy—for example, viewpoint articles, perspective articles and opinion articles. 

**Table 1:** Characteristics of included studies

Author(s) Year	Research approach	Research design	Theory, framework or model	Recruitment Sampling	Data collection	Data analysis
Adjei et al.^83^ 2018	Qualitative	Multifaceted research design	Critical race study	Existing networks Purposive	Semistructured interviews (one-on-one and focus group)	Qualitative analysis
Aziz et al.^82^ 2022	Mixed methods	Cross-sectional study	NA	Local media (newspapers and radio) and community networks (notice boards and social networks) Representative	Surveys (with qualitative, open-ended questions)	Statistical analysis (independent sample *t* tests, ANOVA, compared mean scores), content analysis
Baker et al.^81^ 2001	Qualitative	Constructivist research design	NA	Board members of multicultural associations assisted recruitment Purposive and snowball	Interviews (one-on-one)	Constant comparative analysis
Beagan and Etowa^39^ 2009	Mixed methods	NA	Everyday racism	Word of mouth Nonrepresentative purposive and snowball	Surveys and semistructured interviews	Thematic analysis, statistical analysis
Beagan and Etowa^40^ 2011	Qualitative	Narrative research	NA	Word of mouth Purposive and snowball	In-depth, semistructured interviews	Thematic analysis
Beagan et al.^41^ 2012	Mixed methods	Exploratory design	NA	Word of mouth Purposive and snowball	Interviews and surveys	Thematic analysis, statistical analysis
Beagan and Chapman^42^ 2012	Qualitative	NA	NA	Advertisements and word of mouth Purposive and snowball	Semistructured, one-on-one interviews and observation	Thematic analysis
Beals and Wilson^25^ 2020	Qualitative	Arts based and community-based research	Intersectional Black feminist decolonizing perspectives and identity theory	Through “Proclaiming Our Roots” project Convenience	Workshops, semistructured interviews	Thematic analysis
Bernard et al.^77^ 2014	Mixed methods	Naturalistic inquiry and cross-sectional secondary data analysis	NA	Purposive	Secondary data analysis (survey data) of data from Racism, Violence, and Hate Study and interviews (one-on-one and focus group)	Thematic analysis
Bernard^38^ 2020	Other	NA	Practice Principles based on Africentric theory	NA	NA	NA
Bhawra et al.^79^ 2021	Quantitative	Quasiexperimental (prospective cohort study)	NA	Face-to-face intercept sampling method stratified by region and type of location	Surveys	Statistical analysis (descriptive, multinomial logistic regression)
Crooks^43^ 2018	Qualitative	Case study	NA	NA	NA	NA
Davidson et al.^44^ 2001	Qualitative	Cross-sectional	NA	Database of provincial telephone numbers, systematic sampling procedures from a list of randomly selected Nova Scotian households in the database	Surveys	Statistical analysis (descriptive statistics, one-way ANOVAs, Pearson chi-square tests)
Davis^45^ 1964	Quantitative	Cross-sectional	NA	Students were drawn from Grades 7, 8 and 9 to correspond as closely as possible in educational level to Karon’s school children Purposive	Standardized tests	Statistical analysis
Davis et al.^46^ 2013	Qualitative	NA	Critical race theory	Phase 2: flyers, word of mouth and community networks Purposive	Interviews (one-on-one and focus groups)	Thematic analysis
Delisle and Sweeney^47^ 2018	Other	NA	NA	NA	NA	NA
Etowa^48^ 2005	Qualitative	Grounded theory	NA	Community organizations and existing networks Purposive convenience, theoretical and snowball sampling	Interviews (informal), observation, field notes and group meetings	Constant comparative method
Etowa et al.^49^ 2007	Mixed methods	Participatory action research and interpretive phenomenology	Interpretive phenomenology paradigm (Africentric paradigm)	Purposive sampling	Interviews (one-on-one and focus groups), community workshops and survey	Thematic analysis and statistical analysis (descriptive)
Etowa et al.^50^ 2007	Qualitative	Participatory action research	NA	Community networks Purposive, theoretical and snowball sampling	Interviews (one-on-one)	NA
Etowa et al.^51^ 2007	Mixed methods	Participatory action research	NA	Existing networks of community facilitators and community networks Purposive and snowball sampling	Interviews (one-on-one and focus groups) and surveys	Thematic analysis
Etowa et al.^52^ 2009	Qualitative	Grounded theory	NA	Theoretical sampling	Interviews (one-on-one and focus groups), interview observations and field notes	Constant comparative method
Etowa et al.^53^ 2012	Other	NA	Participatory action research	NA	Surveys	Statistical analysis and thematic analysis
Etowa^54^ 2012	Qualitative	Feminist participatory action research	NA	NA	Interviews (one-on-one and focus groups)	Thematic analysis
Etowa et al.^55^ 2017	Mixed methods	NA	NA	Purposive, snowball sampling	Surveys, interviews (one-on-one and focus groups) and community workshops	Qualitative and quantitative analysis
Evans et al.^56^ 2005	Qualitative	Participatory action research	NA	Community networks (organizations and leaders)	Interviews (focus groups)	Thematic analysis
Gahagan et al.^57^ 2011	Mixed methods	NA	NA	Flyers, existing networks (community and healthcare services contacts)	HIV surveillance data and interviews (in-depth, semistructured, one-on-one)	Thematic analysis and statistical analysis (descriptive)
Jean-Pierre^58^ 2021	Qualitative	Cross-sectional	Cultural trauma	Word-of-mouth, email, posters Snowball sampling/respondent-driven sampling	Interviews (one-on-one and focus groups)	Thematic analysis
Johnston et al.^59^ 2003	Quantitative	Cohort study	NA	Mailing letters Sample derived from a provincial database	Data registry	Statistical analysis (two dimensional cross-tabulations and chi-square tests, logistic regression)
Kisely et al.^60^ 2008	Quantitative	Retrospective cohort study	NA	NA	Administrative databases	Statistical analysis (incidence rates, descriptive statistics)
LeBrasseur^74^ 2022	Mixed methods	Participatory action research	NA	Existing networks (to form advisory committee)	Survey (digitally map-based)	Literature review, descriptive analysis
Maddalena^61^ 2005	Qualitative	Case study, reflexive ethnography	Feminist ethics	Mail survey Purposive sample	Interviews, surveys and document review	Discourse analysis, thematic analysis, reflexive ethnography and ethnography
Maddalena^76^ 2010	Qualitative	Case study	NA	Purposeful sampling	Interviews (one-on-one, in-depth)	Discourse analysis, thematic analysis, reflexive ethnography and ethnography
Maddalena et al.^75^ 2010	Qualitative	Case study	NA	Purposeful sampling	Interviews (one-on-one)	Thematic analysis and discourse analysis
Maddalena et al.^62^ 2013	Qualitative	NA	Naturalistic inquiry and participatory action research	Through local community church Purposeful and snowball sampling	Interviews (focus groups)	Thematic and discourse analysis
Nourpanah^63^ 2019	Qualitative	Ethnography	NA	NA	Interviews (ethnographic)	Narrative analysis
Nyika^64^ 2022	Qualitative	Photovoice	Critical race theory–social constructivism framework	Invitations through school staff (principals, vice principals and support staff) Purposeful	Photovoice and interviews (one-on-one and focus groups)	Thematic analysis
Paynter et al.^65^ 2022	Qualitative	NA	Community-based research	Email to past doula training participants	Semistructured interviews	Thematic analysis
Ristovski-Slijepcevic et al.^67^ 2008	Qualitative	NA	NA	Community based organizations, community contacts and public notices	Interviews, observations	Thematic analysis
Ristovski-Slijepcevic et al.^66^ 2010	Qualitative	NA	NA	Community-based organizations and public notices Purposive and snowball sampling	Part of a family-oriented food study (described elsewhere), interviews, shopping trip, participant observation	Critical discourse analysis
Taylor^80^ 2022	Qualitative	Grounded theory	Straussian grounded theory	Social media, community organization email lists Purposeful and snowball sampling	Interviews	The data were analyzed using techniques specific to Straussian grounded theory
Wade^78^ 1973	Quantitative	Cross-sectional	Maslow’s Theory of Needs	Existing network, letter to school Random sampling	Standardized tests and surveys	Theoretical analysis
Waldron^68^ 2015	Other	NA	NA	NA	Workshops	NA
Waldron^69^ 2018	Other	NA	NA	NA	NA	NA
Waldron^70^ 2018	Other	NA	NA	NA	NA	Sociospatial analysis
Watson^71^ 2009	Qualitative	Ethnography	Interdisciplinary, problem-oriented approach	Direct requests at educational classes	Observations and interviews (one-on-one)	Thematic analysis
Weerasinghe^72^ 2012	Qualitative	NA	Cultural health capital framework	Contact information obtained from community organizations	Surveys, interviews and discussions	Theory-driven coding and inductive coding
Wong et al.^73^ 2005	Other	NA	NA	Project assistants were used for recruitment, existing networks	Interviews (focus groups) and surveys	NA

**Abbreviations:** ANOVA, analysis of variance; HIV, human immunodeficiency virus; NA, not applicable. 

**Note:** “Other” refers to articles that do not fall under the traditional qualitative–quantitative dichotomy—for example, viewpoint articles, perspective articles and opinion articles.

**Target populations**

Varying terminology was used to describe Black populations in Atlantic Canada—for example, ANS,^38^,^40^,^41^,^77^ Black^48^,^66^,^81^ and African Canadian.^75^ Several included studies focussed on multiple populations (e.g. Black populations and Punjabi British Columbians, White Canadians, Southeast Asian immigrants, etc.).^59^,^66^,^67^,^72^,^78^ Studies sampled diverse Black populations at the intersections of gender, age, socioeconomic status, ability and immigration status. While some studies focussed on Black communities,^70^,^73^ most focussed on subpopulations such as middle-aged women,^39^,^40^,^49^,^55^ school-aged youth^45^,^78^ and nurses.^48^,^52^ No studies focussed on children, and four focussed on youth.^45^,^64^,^78^,^81^ Three studies focussed on parents or family entities.^46^,^75^,^83^ With respect to gender, 13 studies focussed solely on women,^39^-^41^,^44^,^48^,^50^,^51^,^53^-^55^,^59^,^71^,^72^ while no studies solely focussed on men, and one study included, but did not focus on, gender-diverse individuals.^25^

***Findings and recommendations of the available research***

**Key findings**

The key outcomes and results from included studies had varying implications for Black health and associated research. The pervasiveness and impact of experiencing racism was threaded through the findings of several studies.^39^,^41^,^48^,^51^,^71^,^81^ For example, racism impacted engagement in education,^81^ had a distinct influence on occupation^39^ and directly impacted health.^51^ Several studies explored conceptualizations of health and health concerns. A core focus of such studies was developing an understanding of the social determinants of health that impact Black Canadians.^46^,^51^,^80^ For example, Etowa and colleagues highlighted key determinants of health for rural Black Nova Scotian women and their families (e.g. racism, poverty, unemployment, access to health services and caregiving roles).^51^ One study explored definitions of health.^53^ Other studies found key health outcomes for Black Canadians, including higher likelihood of living in a food-insecure household^79^ and higher morbidity levels associated with treated disease.^60^

Culture was woven through the findings of several studies. One study noted the importance of meals to ANS as a source of pride and identity.^42^ Culture-based spirituality was also noted as a strong influence on African Canadians in Halifax.^77^ Spirituality and religion were used as key coping mechanisms to deal with racism, as well as means of advocating for social justice.^41^ Furthermore, a lack of access to culturally appropriate, sensitive and safe services and education was highlighted.^58^,^61^,^76^

***Recommendations***

Many studies offered valuable future directions for Black health data. The recommendations broadly fit into five categories: (1) recognizing the pervasive and real impact of racism on Black populations; (2) developing and providing education and cultural competence and safety training; (3) building partnerships with community prior to project start; (4) recognizing the importance of community-based approaches; and (5) developing a stronger research foundation. 

Research conducted with Black populations is fundamentally flawed if the research team does not recognize the impacts and pervasive nature of racism. Etowa and colleagues state that it is important that “research in this area be undertaken with the recognition that race interacts with numerous other variables and experiences to determine the health of Canadian Black women and their families.”^51^^,p.72^ The interactions between race and other variables and experiences denote the pervasive impact of racism on everyday lives of Black people. 

Developing and providing education and cultural competence and safety training was the second most common recommendation. One study recommended ongoing education training for faculty and staff in nursing programs due to a lack of diversity and social inclusion training.^48^ Another study suggested the need for cultural competence and safety training within curricula for childbirth educators and healthcare providers.^54^ Jean-Pierre adopted a systems-level recommendation aimed at integrating culturally relevant and sustaining pedagogy within Nova Scotia’s education system.^58^ Furthermore, she provides an explanation of how such integration would benefit Black learners: 

Implementing culturally relevant and sustaining pedagogy across Nova Scotia may foster equitable learning environments for Black learners by providing fluency in their cultural heritage, fulfilling the long-awaited democratic promise of integrated public schools, and representing a form of civic repair where we redress the legacy of anti-Black racism in education.^58^^,p.1167-8^

Several other studies recommended cultural and structurally appropriate and competent training be implemented within healthcare and health education. Educational recommendations were mostly made for childbirth settings and professionals,^54^,^65^,^71^ followed by nurses.^48^

Partnership and community were at the heart of culturally and structurally competent research throughout this review. This is reflected in the studies that recommended building community partnerships prior to commencing research.^51^,^54^,^62^,^68^,^69^,^73^ Etowa and colleagues suggested that embedding community partnerships as an explicit goal can help research teams fully understand health issues and achieve improved health outcomes for Black populations.^51^ Another study added that partnerships foster mutual respect between healthcare providers or organizations and community that, in turn, empowers communities to find their voices.^54^ While partnerships were a core focus of recommendations to improve Black health research, one study recommended exploring mechanisms of developing partnerships to ensure effective engagement.^62^

***Considerations outside of Eurocentric approaches to research***

**Community engagement**

Community engagement (CE) is a process of working collaboratively with populations or groups that share specific characteristics to positively impact the health and well-being of that population.^84^ CE operates on a spectrum from minimal involvement to community-led initiatives. CE as a spectrum was reflected in the conceptualizations expressed in the included studies in this review. For example, three studies discussed having appropriate population representation on the research team,^42^,^55^,^75^ while others discussed community involvement in varying ways, including having community members assist in recruitment,^48^ connecting with community organizations,^67^ creating community advisory committees^46^,^73^,^74^ and encouraging community leads and/or involvement throughout the entire research process.^51^,^54^,^56^,^75^

Articles often included community engagement but did not discuss the importance of the process. For example, Beagan and Etowa acknowledge the presence of “close-knit local communities”^40^^,p.287^ and developed an African Nova Scotian research team, but do not touch on the reasons for adopting this method of community engagement. Further, Davis and colleagues hired an advisory committee of community members, but again, do not discuss the importance of the process of engaging community members in the research process.^46^ Wong and colleagues touch on a “failure to identify leaders in each of the participant communities to act as cultural representatives”^73^^,p.12^ as part of the reason for difficulty with recruitment and response rates. 

**Cultural considerations**

Connecting with community arose as a key cultural consideration, and the importance of working collaboratively with community is discussed in the community engagement section above. Other key considerations were selecting appropriate theoretical frameworks^50^,^51^,^58^,^68^,^69^,^74^,^75^ and engaging members of the Black population throughout the research cycle.^42^,^52^,^53^,^62^,^72^,^75^ The theoretical frameworks adopted by included studies are outlined in Table 1. It is important to note that certain frameworks were discussed as culturally relevant and appropriate. Specifically, participatory action frameworks were frequently discussed as appropriate and relevant.^50^,^51^,^68^,^69^,^74^ Other frameworks that were included, discussed and identified as culturally considerate were cultural trauma^58^ and storytelling.^75^

## Discussion

This rapid review was conducted as part of a national inquiry across three key regions in Canada (the Prairies, Quebec and the Atlantic region). The purpose of this review was to develop an understanding of the research cycle in the field of Black health in the Atlantic provinces of Canada. Specifically, key issues, opportunities and promising practices were identified to further improve the state of Black health research across the region. The results of our study suggest a dearth of literature in the Atlantic region, but also key areas for consideration to ensure that research is conducted with Black Canadians in a manner that is safe, culturally appropriate and beneficial to the community.

The lack of available literature (only 47 studies) poses a serious concern to ensuring culturally and structurally appropriate research. Trends in number of publications by year indicate that the turn of the millennium sparked an increased interest in Black health. However, the International Decade for People of African Descent—declared by the United Nations to extend from 2015 to 2024—did not result in a further, noticeable increase in interest in and generation of Black health data.^85^ Because of the prevalence of anti-Black racism and systemic injustice, a broader foundation for this research area should be available. 

A recent scoping review of participatory research methods in Indigenous health research supports this notion.^86^ The study identified 211 articles for inclusion. Given the similarities in proportion of the population that Indigenous and Black individuals represent, and similarities in scope between the two reviews, one might expect a greater availability of research on Black health. The sparsity of available evidence may, in part, be due to the lack of mandatory race-based data collection in Canada,^87^ community fatigue from exploitative research projects or a lack of culturally and structurally appropriate and ethical training for researchers and practitioners.^48^,^54^,^58^

Central to contributing to a broader foundation for Black health research in Atlantic Canada is ensuring that the design, development and execution of research is premised on cultural considerations. Our findings suggest that community engagement is an integral consideration for conducting research with Black populations and may improve project outcomes. Husbands and colleagues found that through engaging Black churches in an intervention (Black PRAISE) to promote critical awareness of HIV, congregants’ knowledge of HIV increased.^88^ In addition to the importance of community leaders discussed by Wong and colleagues,^73^ Black PRAISE may indicate the how crucial context is in the process of community engagement with Black populations. Integrating community engagement techniques into research has the potential to ensure culturally and structurally competent research is undertaken with Black populations in Atlantic Canada. 

While community-based and participatory action research were frequently adopted, the importance of community extends beyond the adoption of a model or framework. Data governance frameworks exist that help to explain the centrality of community in Black health data. For example, the Engagement, Governance, Access and Protection (EGAP) Framework, out of Ontario, outlines engagement as the first of four core principles for data governance.^89^ Engagement is not only a process of listening but rather ensuring the project hinges on what is meaningful to communities. This review highlights the many meaningful ways community can be engaged, from the creation of community advisory committees to the inclusion of Black researchers at the beginning of the project. Given the geographic spread and diversity of Black populations in Canada, there is a need for a framework that extends beyond Ontario and that represents the collective data ownership, governance and accessibility rights of all Black Canadians.

This review highlighted the many recommendations arising from the limited Black health research conducted in the Atlantic region of Canada. In order to develop an evidence base that informs action to address health inequities, there is a fundamental need for greater education and consideration of the histories and contributions of Black populations to the region. Currently, the Tri-Council Policy Statement “Ethical Conduct for Research Involving Humans (TCPS 2)” does not offer a module for conducting research with Black Canadians.^90^ We recommend, to ensure and support the implementation of cultural and structural competence and safety training into services, that the federal funding agencies develop an appropriate module, created by Black communities and researchers, that will form the ethical groundwork for all research involving Black participants in Canada. 

***Strengths and limitations***

A notable strength of this study is the rigorous search strategy adopted. The collaboration with a university librarian ensured the search strategy was comprehensive and accurate and captured all applicable and available information on the topic. Another strength is the use of a broad definition of health, enabling the capture of culturally and structurally appropriate and ethical Black health research that specific definitions might overlook. 

This study also has potential limitations. Critical appraisal of literature may be of benefit to assess the trustworthiness of the study findings. Given the importance of community engagement in conducting research with Black populations, a search of grey literature might have identified key community-level data collection initiatives that exist outside the realm of published literature. 

## Conclusion

Findings from this study suggest there is a need for dedicated resources (e.g. a TCPS 2 chapter on ethical conduct in research with Black populations, and regional ethics frameworks for Black populations similar to the EGAP framework) to improve the state of Black health research in Atlantic Canada. There is a paucity of Black health data in the region, particularly for subpopulations including men, children and Black populations in NB, PE and NL. To maximize research participation and outcomes, particular attention should be paid to community engagement throughout the stages of the research process. A nationwide data collection and governance framework would provide an opportunity to improve the state of Black health data in the Atlantic region.

## Acknowledgements

Thank you to the Public Health Agency of Canada for providing the funding to conduct this project. The research team would like to acknowledge Courtney Svab, a librarian intern, for their contributions to the development of the search strategy. We would also like to thank Melissa Rothfus, a university librarian, for providing supervision and expertise in the development of the search strategy for this review. Further, we thank Nicholas Hickens and Shirley Hodder, research assistants, for their contributions to the manuscript draft. 

## Conflicts of interest

This study was funded by the Public Health Agency of Canada. 

## Authors’ contributions and statement

JY, ESC, KJ, BB, CS: data curation.

JY, ESC, KJ, BB, CS: formal analysis.

BHH: funding acquisition.

ESC, KJ, BHH: methodology.

JY, BHH: project administration.

JY, CS: visualization.

JY, ESC, KJ, BB, BHH: writing—original draft.

JY, BHH: writing—review and editing.

The content and views expressed in this article are those of the authors and do not necessarily reflect those of the Government of Canada.
